# Heterogeneity of proangiogenic features in mesenchymal stem cells derived from bone marrow, adipose tissue, umbilical cord, and placenta

**DOI:** 10.1186/s13287-016-0418-9

**Published:** 2016-11-10

**Authors:** Wen Jing Du, Ying Chi, Zhou Xin Yang, Zong Jin Li, Jun Jie Cui, Bao Quan Song, Xue Li, Shao Guang Yang, Zhi Bo Han, Zhong Chao Han

**Affiliations:** 1 The State Key Laboratory of Experimental Hematology, Institute of Hematology and Hospital of Blood Disease, Chinese Academy of Medical Science & Peking Union Medical College, No. 288, Nanjing Road, Heping District, Tianjin, 300020 China; 2 Beijing Institute of Health and Stem Cells, No. 1, Kangding Road, BDA, Beijing, 100176 China

**Keywords:** Mesenchymal stem cells, Bone marrow, Adipose tissue, Umbilical cord, Placental chorionic villi, Pro-angiogenic features, Heterogeneity

## Abstract

**Background:**

Mesenchymal stem cells (MSCs) have been widely proven effective for therapeutic angiogenesis in ischemia animal models as well as clinical vascular diseases. Because of the invasive method, limited resources, and aging problems of adult tissue-derived MSCs, more perinatal tissue-derived MSCs have been isolated and studied as promising substitutable MSCs for cell transplantation. However, fewer studies have comparatively studied the angiogenic efficacy of MSCs derived from different tissues sources. Here, we evaluated whether the in-situ environment would affect the angiogenic potential of MSCs.

**Methods:**

We harvested MSCs from adult bone marrow (BMSCs), adipose tissue (AMSCs), perinatal umbilical cord (UMSCs), and placental chorionic villi (PMSCs), and studied their “MSC identity” by flow cytometry and in-vitro trilineage differentiation assay. Then we comparatively studied their endothelial differentiation capabilities and paracrine actions side by side in vitro.

**Results:**

Our data showed that UMSCs and PMSCs fitted well with the minimum standard of MSCs as well as BMSCs and AMSCs. Interestingly, we found that MSCs regardless of their tissue origins could develop similar endothelial-relevant functions in vitro, including producing eNOS and uptaking ac-LDL during endothelial differentiation in spite of their feeble expression of endothelial-related genes and proteins. Additionally, we surprisingly found that BMSCs and PMSCs could directly form tubular structures in vitro on Matrigel and their conditioned medium showed significant proangiogenic bioactivities on endothelial cells in vitro compared with those of AMSCs and UMSCs. Besides, several angiogenic genes were upregulated in BMSCs and PMSCs in comparison with AMSCs and UMSCs. Moreover, enzyme-linked immunosorbent assay further confirmed that BMSCs secreted much more VEGF, and PMSCs secreted much more HGF and PGE2.

**Conclusions:**

Our study demonstrated the heterogeneous proangiogenic properties of MSCs derived from different tissue origins, and the in vivo isolated environment might contribute to these differences. Our study suggested that MSCs derived from bone marrow and placental chorionic villi might be preferred in clinical application for therapeutic angiogenesis.

**Electronic supplementary material:**

The online version of this article (doi:10.1186/s13287-016-0418-9) contains supplementary material, which is available to authorized users.

## Background

Mesenchymal stem cells (MSCs) are able to self-renew and differentiate into a lineage of mesenchymal cells, such as adipocytes, osteocytes, and chondrocytes. Besides, they are of low immunogenicity and can exert potent immunosuppression on HLA-mismatched PBMCs, which make them ideal candidates for cell therapy and tissue engineering [1]. Moreover, MSCs have been widely reported to exert angiogenesis in in-vitro and in-vivo experiments [2]. Also, human bone marrow-derived MSCs (BMSCs) have been proven effective in clinical application for therapeutic angiogenesis in patients with critical limb ischemia [3]. Because of their favorite angiogenic properties, MSCs are attractive in clinical cell therapy and tissue engineering for ischemia disease treatment [4].

MSCs were first isolated from bone marrow in 1976 [5], and other tissue-derived MSCs were then later harvested in succession. During the last decades, MSCs derived from perinatal tissues [6], including umbilical cord blood, umbilical cord, and placenta, have attracted attention because of their noninvasive isolation methods and minimal ethical issues. Moreover, perinatal tissue-derived MSCs are young cells without higher possibilities of incorporated mutation in comparison with adult tissue-derived MSCs [6].

Many studies have comparatively analyzed the differential properties and biological functions of MSCs derived from perinatal and adult tissues, including their molecule profile [7], tridifferentiation potentials [8], proliferation/clonogenic formation capacities [9], immunomodulatory functions [10], and hematopoietic support abilities [11]. However, fewer studies have so far investigated their angiogenic differences. Previous studies have shown that both adult BMSCs and adipose tissue-derived MSCs (AMSCs) could induce remarkable therapeutic angiogenesis [12]. Umbilical cord-derived MSCs (UMSCs) and placental chorionic villi-derived MSCs (PMSCs) have also been reported to display angiogenic activity in in-vitro and in-vivo experiments [13]. However, under the same culture conditions, do these perinatal MSC populations have similar angiogenic effects as the typical adult BMSCs and AMSCs? Does the in-situ environment of MSCs affect their angiogenic properties? Is there any influence of the development stage on their angiogenic functions? To answer these questions, we designed this project and comparatively analyzed the angiogenic differences of BMSCs, AMSCs, UMSCs, and PMSCs.

## Methods

### Cell culture

The study was approved by the Ethical Committee and the Institutional Review Board of the Chinese Academy of Medical Science & Peking Union Medical College. Bone marrow was collected from healthy volunteers in the Blood Diseases Hospital. BMSCs were isolated following the protocol described previously [14]. AMSCs, UMSCs, and PMSCs at passage 1 were supplied by the Cell Products of National Engineering Research Center (http://www.amcellgene.com) [15, 16]. Additionally, one pair of UMSCs and PMSCs that derived from the same donor was used. All volunteers provided informed consent. BMSCs, AMSCs, UMSCs, and PMSCs were cultured in the same standard culture condition. The standard complete culture medium for MSCs was DMEM/F12 (DF12; Gibco, Grand Island, NY, USA), 10 % fetal bovine serum (FBS; HyClone), 2 mM glutamine (Sigma, St. Louis, MO, USA), 100 U/ml penicillin-streptomycin (P/S; Invitrogen, Carlsbad, CA, USA), and 10 ng/ml epidermal growth factor (EGF; Peprotech). Human umbilical vein endothelial cells (HUVECs) were harvested by digesting umbilical cord vein for 15 minutes at 37 °C. HUVECs were cultured in Endothelial Growth Medium-2MV (EGM-2MV; Lonza, Walkersville, MD, USA), and HUVECs at passage 3~5 ﻿ were used for experiments.

### Endothelial differentiation

Cells were seeded on Matrigel (1:100 dilution; BD Bioscience, Bedford, MA, USA) precoated flasks, and cultured with EGM2-MV supplemented with 50 ng/ml VEGF (Prepotech) [17] for 2 weeks. Endothelial differentiation medium was changed twice a week. Cells cultured in MSC complete medium were used as the control. Cells with or without endothelial differentiation were then harvested, and the expression of endothelial-related genes was independently tested by RT-PCR and immunostaining.

### Real-time PCR

Endothelial-related genes were respectively tested on undifferentiated and differentiated MSCs, and their relative expression level in undifferentiated MSCs was normalized to 1; thus the fold-change in gene expression of differentiated MSCs was calculated as 2^–△△CT^. Additionally, to comparatively analyze the expression level of angiogenic cytokines on BMSCs, AMSCs, UMSCs, and PMSCs, RT-PCR was also performed. The relative expression level of the candidate gene in particular BMSCs or PMSCs was normalized to 1, and its relative expression fold in other cells was shown as 2^–ΔΔCT^. The real-time PCR was performed as follows: total RNA was extracted using the E.Z.N.A. Total RNA Kit I (OMEGA, Norcross, GA, USA), and cDNA was then synthesized by using the MLV RT Kit (Invitrogen). The SYBR Green detection method was employed, and the Applied Bio system 7900 or 7300 Real-Time PCR System (Foster City, CA, USA) was used. Each sample was performed in triplicate (*n* = 3–5). Primers involved are listed in Additional file 1: Table S1.

### Immunostaining

EC-differentiated MSCs were harvested and seeded at 2 × 10^4^ cells/cm^2^ in glass-bottom cell culture dishes (NEST) overnight. Cells were first fixed with 4 % paraformaldehyde (PFA) for 15 minutes. After permeabilization with 0.25 % Triton X-100 solution (Sigma) for 30 minutes, the nonspecific epitope of cells was blocked by 0.2 % albumin bovine V for 30 minutes. The cells were then incubated with mouse anti-human von Willebrand Factor (vWF, 1:100 dilution; Abcam, Cambridge, MA, USA) or rabbit anti-human eNOS antibodies (1:100 dilution; Abcam) for 60 minutes, followed by staining with FITC-conjugated anti-rabbit or anti-mouse antibodies (1:100 dilution; Invitrogen, Molecular Probes) for 30 minutes. Cells stained with FITC-conjugated anti-mouse or anti-rabbit IgG alone served as the control. Finally, cells were stained with DAPI for 5 minutes, fixed with 2 % PFA, and then photographs were taken by PerkinElmer UltraVIEW Vox confocal microscope (PerkinElmer, Waltham, MA, USA) under × 200 magnification.

### Acetylated low-density lipoprotein-uptaking assay

To assess whether cells after endothelial differentiation developed the endothelial-specific function in terms of uptaking acetylated low-density lipoprotein (ac-LDL), the acLDL-uptaking assay was performed. We harvested EC-differentiated MSCs and seeded them on small culture dishes with a glass bottom (NEST). After overnight incubation in serum-free culture medium, cells were cultured in fresh complete medium with 10 μg/ml Dil-acLDL (Invitrogen) for 4 hours. Later, cells were washed twice with PBS and fixed with 4 % PFA following DAPI staining. HUVECs were used as positive control. Photographs were taken using a PerkinElmer UltraVIEW Vox confocal microscope.

### Conditioned medium preparation

MSCs were plated at 4 × 10^4^ cells/cm^2^ in a T25 flask overnight. After washing twice with PBS, cells were cultured with 10 ml EBM2 (Lonza) for another 2 days. Their conditioned media (CMs) were then collected, centrifuged at 400 × *g* for 10 minutes to remove the cell debris, filtered through a 0.2 μm filter (Pall Corporation, Ann Arbor, MI, USA), and frozen at –80 °C for further studies. MSCs derived from three donors were used.

### In-vitro Matrigel tube formation assay

#### Direct Matrigel tube formation assay

To investigate their angio-vasculogenic capacities [18], BMSCs, AMSCs, UMSCs, and PMSCs were collected and seeded directly on a Matrigel (BD Bioscience) precoated 96-well plate at 2 × 10^4^ cells/well in MSC complete medium. Photographs were taken using the microscope (Olympus, Melville, NY, USA) after 12 hours of incubation (scale bar = 500 μm). Tube numbers in each well were counted and each sample was performed in triplicate (BMSCs, *n* = 2; AMSCs, UMSCs, and PMSCs, *n* = 3).

#### Indirect Matrigel tube formation assays

To better study the paracrine action of BMSCs, AMSCs, UMSCs, and PMSCs, we used their CMs to incubate endothelial cells and further assessed their trophic effects on the angiogenic function of endothelial cells. CMs supplemented with 2 % FBS, EBM2 supplemented with 2 % FBS (served as the negative control), and EGM2-MV (contained plentiful cytokines and 2 % FBS, served as the positive control) were used to culture endothelial cells for 9 hours, respectively. Endothelial cells were seeded on Matrigel at 2 × 10^4^ cells/well in a 96-well plate. Photographs were taken by microscope (scale bar = 500 μm). Tube numbers in each well were counted, and the total tube length and total tube area in each well were measured using ImageJ software (NIH, USA). Three donor-derived CMs were used and each sample was performed in duplicate.

### Endothelial cell proliferation assay

To investigate the proproliferative effects of the secretion of BMSCs, AMSCs, UMSCs, and PMSCs, endothelial cells were incubated with different MSC population-derived CMs. The endothelial cell proliferation was measured using the Cell Counting Kit-8 (Dojindo, Rockville, MD, USA). Endothelial cells were first seeded at 1.0 × 10^4^ cells/well in a 96-well plate overnight. After gently removing the medium, cells were washed twice with PBS, and then CMs supplemented with 2 % FBS, EBM2 supplemented with 2 % FBS (served as the negative control), and EGM2-MV (served as the positive control) were added to the cells for another 48 hours. Each sample was performed in triplicate. ΔOD450 indicated the final data after subtracting the background.

### Enzyme-linked immunosorbent assay

To determine the concentration of VEGF, HGF, bFGF, and PGE2 in supernatants of MSCs, enzyme-linked immunosorbent assays (ELISAs) were performed. The 48-hour supernatants of MSCs with an initial seeding density of 20,000 cells/well in a 96-well plate were collected for VEGF, HGF, and bFGF measurements, while the 72-hour supernatants of MSCs with an initial density of 10^5^ cells/well in a six-well plate were collected for PGE2 measurements. All of the supernatants were centrifuged at 400 × *g* for 10 minutes and then measured by their corresponding ELISA kits. The ELISA kits for VEGF, HGF, and bFGF were purchased from Neobioscience Biotech (Shenzhen, China), and the PGE2 ELISA kit was purchased from Cayman Chemicals. All of the procedures strictly followed the corresponding instructions. Supernatants derived from three donors were used.

### Statistical analysis

Statistical analysis was performed by GraphPad Prism 6.0 software (Graph Pad Software, Inc., San Diego, CA, USA). All data are shown as the mean ± SEM. One-way ANOVA followed by Bonferroni multiple comparisons was employed to determine the statistical significance. Paired *t* test was used to analyze the endothelial gene modification after endothelial differentiation. The result was considered statistically significant if *p* < 0.05.

## Results

### Limited potentials of endothelial differentiation

**Fig. 1:**
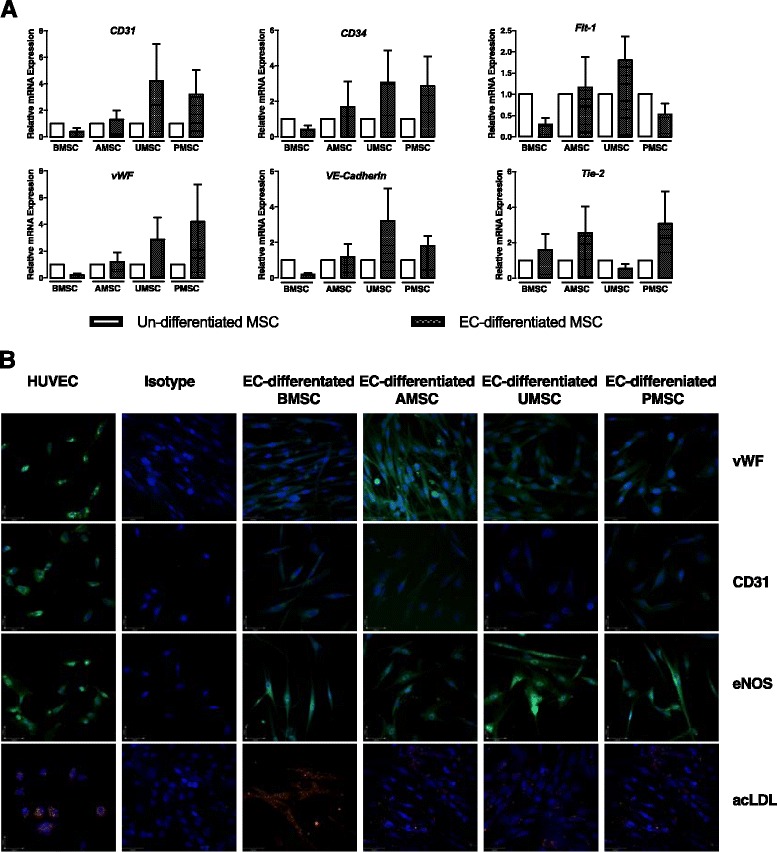
Endothelial differentiation potential of different MSC populations is heterogeneous and limited. **a** Relative expression levels of *CD31*, *CD34*, *Flt1*, *vWF*, *VE-cadherin*, and *Tie-2* were investigated in undifferentiated and EC-differentiated BMSCs, AMSCs, UMSCs, and PMSCs. The candidate gene expression in undifferentiated MSCs was normalized to 1, and the relative fold-change in the corresponding EC-differentiated cells was shown as 2^–ΔΔCT^. BMSCs derived from two donors, and AMSCs, UMSCs, and PMSCs derived from three individuals were used. **b** Confocal microscope was used to investigate the expression of vWF and CD31 in EC-differentiated MSCs and HUVECs. To identify whether the EC-differentiated cells displayed similar functions to endothelial cells, immunostaining of eNOS and acLDL-uptaking assay were respectively performed. All photographs were captured at × 200 magnification. HUVECs served as positive control. *MSC* mesenchymal stem cells, *HUVEC* human umbilical vein endothelial cells, *BMSC* bone marrow-derived MSCs, *AMSC* adipose tissue-derived MSCs, *UMSC* umbilical cord-derived MSCs, *PMSC* placental chorionic villi-derived MSCs, *vWF* von Willebrand Factor, *eNOS* endothelial nitric oxide synthase, *acLDL* acetylated-low density lipoprotein

Previous studies have shown that the endothelial differentiation mechanism participates in MSC-mediated wound healing [19]. To investigate their capabilities of endothelial differentiation, BMSCs, AMSCs, UMSCs, and PMSCs were harvested and induced into endothelial cells in vitro for 2 weeks. The undifferentiated and differentiated MSCs were then collected, and the endothelial gene expression further comparatively analyzed. Data revealed that the relative expression levels of *CD31*, *CD34*, *Flt1*, *vWF*, *VE-Cadherin* (*VEC*), and *Tie-2* were altered differently in EC-differentiated MSCs in comparison with undifferentiated cells; however, no statistical significance was found (*p* > 0.05) (Fig. 1a). There was an increase in expression of *CD31*, *CD34*, *vWF*, and *VEC* in EC-differentiated AMSCs, UMSCs, and PMSCs but a decreased expression in EC-differentiated BMSCs. Similarly, *Flt-1* was upregulated in AMSCs and UMSCs but declined in BMSCs and PMSCs after endothelial differentiation. *Tie-2* expression was raised to various degrees in BMSCs, AMSCs, and PMSCs during endothelial differentiation, but with a falloff in UMSCs. To better define the expression of endothelial-related proteins and the unique functions of cells after endothelial differentiation, an immunostaining assay [20, 21] and an acLDL-uptaking assay [22] were performed respectively (Fig. 1b). Our data showed that EC-differentiated MSCs weakly expressed vWF and CD31 in contrast to the HUVECs (positive control). However, MSCs produced eNOS and developed acLDL uptaking capacities to some extent after endothelial differentiation, which were special functions of endothelial cells. This observation indicated that MSCs could develop some properties of endothelial cells under appropriate conditions.

### Heterogeneous angio-vasculogenic capacities of BMSCs, AMSCs, UMSCs, and PMSCs on in-vitro Matrigel tube formation assay

**Fig. 2:**
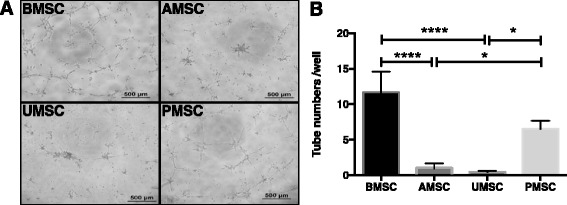
MSCs derived from different tissue sources display distinct tube formation capacities on Matrigel in vitro. MSCs were seeded on Matrigel in vitro at 2 × 10^4^ cells/well. MSCs could form tube-like structures on Matrigel temporarily (**a**). Representative photographs were taken after 12 hours of incubation (*scale bar* = 500 μm). **b** Numbers of tube structures were counted and analyzed (BMSC, *n* = 2; AMSC, UMSC, and PMSC, *n* = 3). Each sample was performed in triplicate (*p < 0.05, ****p < 0.0001). *MSC* mesenchymal stem cells, *BMSC* bone marrow-derived MSCs, *AMSC* adipose tissue-derived MSCs, *UMSC* umbilical cord-derived MSCs, *PMSC* placental chorionic villi-derived MSCs

The in-vitro Matrigel tube formation assay was generally used as the first screen to test whether a compound might participate in angiogenesis [23]. MSCs were reported to spontaneously generate 3-D capillary-like structures on Matrigel in vitro [18, 24]. To determine their angio-vasculogenic capacities, BMSCs, AMSCs, UMSCs, and PMSCs were directly seeded on Matrigel and the tube formation was observed after 12 hours of incubation. Interestingly, intact tube structures were seen in the BMSC and PMSC groups rather than in the groups of AMSCs and UMSCs (Fig. 2a). Tube numbers in the BMSC and PMSC groups were 11.65 ± 2.92 and 6.49 ± 1.18, respectively, much higher than those in the AMSC and UMSC groups (0.91 ± 0.76 and 0.41 ± 0.20, *p* < 0.05) (Fig. 2b). These observations indicated that BMSCs and PMSCs had better angio-vasculogenic capacities in comparison with UMSCs and AMSCs.

### BMSCs and PMSCs displayed potent paracrine actions on endothelial cells

**Fig. 3:**
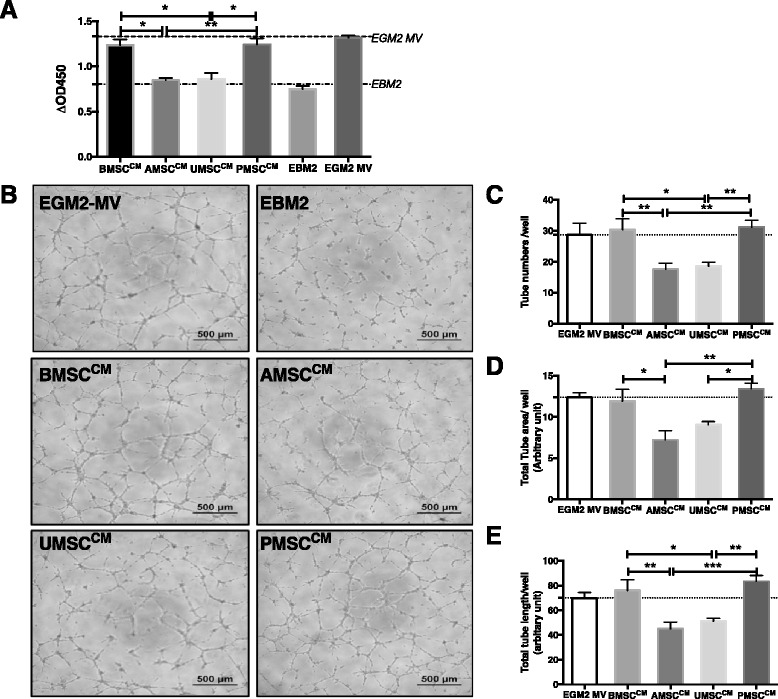
Conditioned medium (*CM*) of BMSCs, AMSCs, UMSCs, and PMSCs exerts various proangiogenic effects on endothelial cells in vitro. CMs were generated from 10^6^ cells after 48 hours of incubation with EBM2. CMs supplemented with 2 % FBS, EBM2 supplemented with 2 % FBS (negative control), and EGM2-MV (containing 2 % FBS, positive control) were used to culture endothelial cells and further comparatively analyze their effects on endothelial cell proliferation. **a** Similar to EGM2-MV, BMSC^CM^ and PMSC^CM^ significantly promoted endothelial cell proliferation in comparison with EBM2 after 48 hours of culture; however, AMSC^CM^ and UMSC^CM^ did not produce such a promotion effect. Each sample was performed in triplicate (*n* = 3, *p < 0.05, **p < 0.01). To determine the bioactivity of secreted factors from MSCs on endothelial cells, an in-vitro Matrigel tube formation assay was performed. Representative photographs were taken after 9 hours of incubation (*scale bar* = 500 μm). **b** Number of tube structures (**c**) was counted; total tube area (**d**) and total tube length (**e**) in each well were measured and analyzed using ImageJ software. Each sample was performed in duplicate (*n* = 3, *p < 0.05, **p < 0.01, ***p < 0.001). *MSC* mesenchymal stem cells, *BMSC* bone marrow-derived MSCs, *AMSC* adipose tissue-derived MSCs, *UMSC* umbilical cord-derived MSCs, *PMSC* placental chorionic villi-derived MSCs

Secreted factors from MSC populations have been reported to significantly enhance the proliferation and function of endothelial cells in vitro [25]. To comparatively analyze their paracrine actions, we respectively used BMSC^CM^, AMSC^CM^, UMSC^CM^, and PMSC^CM^ to culture endothelial cells and comparatively analyzed the effects on the proliferation and tube formation capacity of endothelial cells. We found that BMSC^CM^ and PMSC^CM^ could significantly promote endothelial cell proliferation (1.23 ± 0.06 and 1.24 ± 0.06) in contrast to AMSC^CM^ and UMSC^CM^ (0.84 ± 0.02 and 0.85 ± 0.07; *n* = 3, *p < 0.05 and **p < 0.01) during 48 hours of incubation. The proproliferative effects of BMSC^CM^ and PMSC^CM^ were similar to EGM2-MV (the complete endothelial cell culture medium containing plentiful growth factors), and much greater than EBM2 (0.74 ± 0.03, *p* < 0.01). However, AMSC^CM^ and UMSC^CM^ did not reveal such promotion on the mitosis of endothelial cells compared with the EBM2 group (*n* = 3, *p* > 0.05). Hence, we supposed that BMSCs and PMSCs secreted many more proproliferative factors than AMSCs and UMSCs (Fig. 3a).

In addition, we used BMSC^CM^, AMSC^CM^, UMSC^CM^, and PMSC^CM^ to culture endothelial cells to test whether the secreted factors would affect the tube formation capacities of endothelial cells on Matrigel in vitro. Interestingly, CMs significantly promoted tube formation of endothelial cells by contrast with EBM2, in which no intact tube structure was observed (Fig. 3b). Similarly, we found BMSC^CM^ and PMSC^CM^ significantly promoted endothelial cells to generate intact tube structures, which numbered 30.33 ± 3.59 and 31.17 ± 2.18, respectively, much greater than those in the group of AMSC^CM^ and UMSC^CM^ (17.50 ± 2.09 and 18.50 ± 1.38; *n* = 3, *p < 0.05 and **p < 0.01) (Fig. 3c). The total tube area and total tube length were also greater in the BMSC^CM^ and PMSC^CM^ groups (BMSC^CM^: 11.90 ± 1.46 and 75.93 ± 8.83; PMSC^CM^: 13.39 ± 0.70 and 83.36 ± 4.73) than in the AMSC^CM^ and UMSC^CM^ groups (AMSC^CM^: 7.20 ± 1.12 and 45.05 ± 5.39; UMSC^CM^: 9.03 ± 0.39 and 51.16 ± 2.36; *n* = 3, *p < 0.05, **p < 0.01 and ***p < 0.001) (Fig. 3d, e). These results indicated that the secretion of BMSCs and PMSCs contained more bioactive factors than that of AMSCs and UMSCs in the enhancement of the vasculogenic functions of endothelial cells.

### PMSCs and BMSCs expressed higher levels of angiogenic factors

**Fig. 4:**
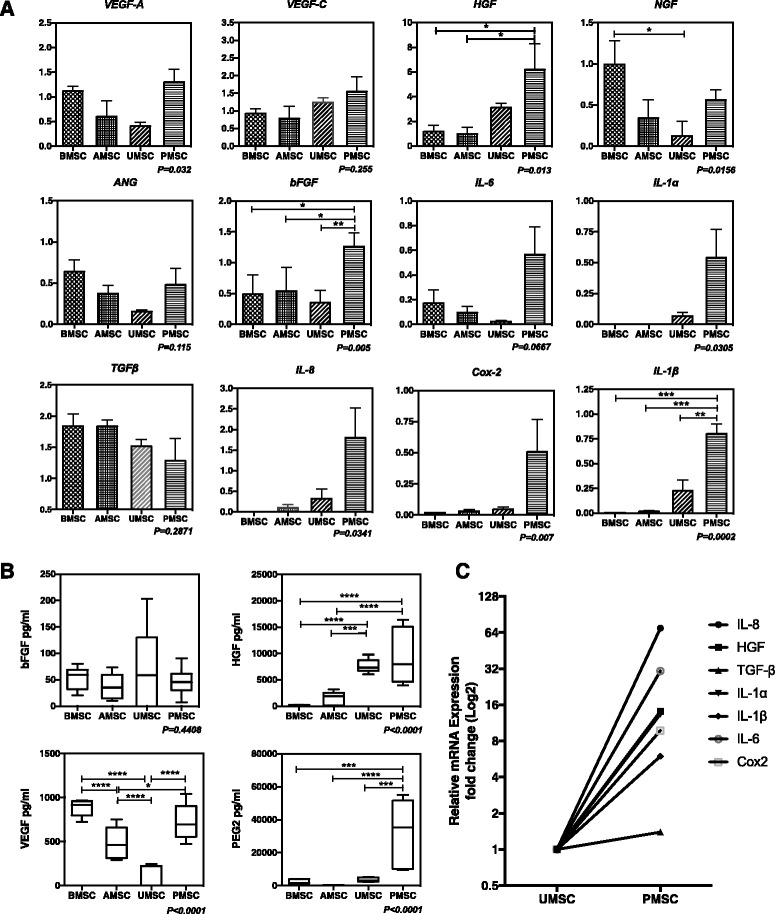
Gene and protein expression of selected paracrine factors in different MSC populations. **a** Relative mRNA expression of selected angiogenic cytokines was assessed, including *VEGF-A*, *VEGF-C*, *HGF*, *bFGF*, *NGF*, *ANG*, *TGF-β*, *IL-6*, *IL-8*, *IL-1α*, *IL-1β*, and *Cox-2*. The relative expression level of the candidate cytokine in one sample of BMSCs or PMSCs was normalized to 1, and all other samples for corresponding gene expression were shown as 2^–ΔΔCT^ fold-change. Each sample was tested in triplicate (*n* = 3–5). **b** Concentrations of bFGF, VEGF, and HGF in 48-hour supernatants and the PGE2 concentration in 72-hour supernatants of BMSCs, AMSCs, UMSCs, and PMSCs were measured using corresponding ELISA kits (*n* = 3, *p < 0.05, **p < 0.01). **c** The relative mRNA expression of *IL-8*, *HGF*, *TGF-β*, *IL-1α*, *IL-1β*, *IL-6*, and *Cox-2* was separately tested on one donor-derived UMSC and PMSC. The relative expression level of the candidate gene in UMSCs was normalized to 1, and the fold-change in PMSCs was expressed as 2^–ΔΔCT^. To better show the different expression of angiogenic genes in UMSCs and PMSCs, data were shown in log_2_. *MSC* mesenchymal stem cells, *BMSC* bone marrow-derived MSCs, *AMSC* adipose tissue-derived MSCs, *UMSC* umbilical cord-derived MSCs, *PMSC* placental chorionic villi-derived MSCs, *VEGF* vascular endothelial growth factor, *HGF* hepatocyte growth factor, *bFGF* basic fibroblast growth factor, *NGF* nerve growth factor, *ANG* angiogenin, *TGF-β* transforming growth factor beta, *IL* interleukin

Cytokines secreted by MSCs played vital roles in the process of MSC-mediated angiogenesis [26]. Referring to the previous research [25, 26], we selected a panel of candidates, including *VEGF-A*, *VEGF-C*, *HGF*, *bFGF*, *NGF*, *angiogenin* (*ANG*), *TGF-β*, *IL-6* [27], *IL-8* [28], *IL-1α* [29], *IL-1β* [30], and *Cox-2* [31], and tested their relative expression levels in different MSC populations. One-way ANOVA analysis revealed that the comparison of *VEGF-A*, *HGF*, *bFGF*, *NGF, IL-6*, *IL-8*, *TGF-β*, *IL-1α*, *IL-1β*, and *Cox-2* expression among different MSC types was statistically significant (*p* < 0.05); while the expression of *VEGF-C*, *ANG*, and *TGF-β* in different MSC populations showed no significant difference (*p* > 0.05). Of note, the relative expression levels of *HGF*, *bFGF*, *IL-6*, *IL-8*, *IL-1α*, *IL-1β*, and *Cox-2* in PMSCs were comparatively higher than that in other MSC populations*.* Besides, BMSCs expressed a comparatively higher level of *VEGF-A*, *NGF*, and *ANG* (Fig. 4a). Therefore, PMSCs, as well as BMSCs, were superior to AMSCs and UMSCs for angiogenic gene expression.

As previous studies have reported, bFGF, VEGF, HGF, and PGE2 could significantly promote angiogenesis [32, 33]. To further explore the secretion of angiogenic-related factors, we assessed the concentrations of bFGF, VEGF, HGF, and PGE2 in supernatants of different MSC populations via ELISA. Data showed that MSCs secreted a lower level of bFGF under normal conditions, which was about 36–69 pg/ml. The concentrations of VEGF in the supernatants of BMSCs and PMSCs were 882.7 ± 55.27 pg/ml and 721.4 ± 120.4 pg/ml, respectively, much higher than that in UMSC-derived supernatants (77.16 ± 77.16 pg/ml; *n* = 3, *p* < 0.01), and a medial level of VEGF (486.3 ± 111.8 pg/ml) was found in AMSC supernatant. Furthermore, the HGF concentration in supernatants of perinatal tissue-derived MSCs (PMSCs: 9466 pg/ml; UMSCs: 7694 pg/ml) was significantly higher than that in adult tissue-derived MSCs (BMSCs: 119 pg/ml; AMSCs: 1589 pg/ml) (*n* = 3, *p* < 0.05; Fig. 4b), which was in accordance with our RT-PCR results and also agreed with the study by Amable et al. [34]. Additionally, the PGE2 concentration in supernatants of PMSC (32,628 pg/ml) was significantly higher than that in other MSC types (BMSC: 2074 pg/ml, AMSC: 337.9 pg/ml; UMSC: 3603 pg/ml; *n* = 3, *p* < 0.05).

To further study whether the in-situ environment would affect the angiogenic properties of MSCs, we evaluated the relative expression of *IL-8*, *HGF*, *TGF-β*, *IL-1α*, *IL-1β*, *IL-6*, and *Cox-2* in one donor-derived UMSC and PMSC by RT-PCR. Our results indicated that PMSCs expressed a remarkably higher level of *IL-8*, *HGF*, *IL-1α*, *IL-1β*, *IL-6*, and *Cox-2* than UMSCs (Fig. 4c). Thus, considering the possible impact of their different isolated environment, placental chorionic villi would be a favorite tissue source for isolating MSCs with superior angiogenic cytokine expression.

## Discussion

In the present study, we performed a parallel comparison of angiogenic potentials of MSCs derived from adult tissues (bone marrow and adipose tissue) and perinatal tissues (umbilical cord and placental chorionic villi). Cells were isolated from different in-situ environments as well as distinct developmental stages, which we hypothesized might affect their angiogenic properties. After verifying the phenotype and trilineage differentiation, we comparatively studied their angiogenic properties from two aspects: endothelial differentiation and paracrine activity. MSCs regardless of their tissue origins have limited capacities to transform into endothelial cells; however, the CM of BMSCs and PMSCs showed stronger proangiogenic activities on endothelial cells in vitro than that of AMSCs and UMSCs.

The endothelial differentiation capacity of MSCs remains controversial. Aguilera et al*.* [20] demonstrated that Wharton’s jelly-derived mesenchymal cells expressed high levels of endothelial markers (CD31 and KDR) and also synthesized NO after 30 days of endothelial differentiation. Additionally, Ikhapoh et al. [35] and Benavides et al. [21] respectively reported that BMSC and amniotic fluid-derived stem cells were capable of differentiation into endothelial cells. By contrast, Choi et al. [36] reported that Wharton’s jelly-derived mesenchymal stromal/stem cells did not express endothelial specific markers (VEGFR2, Tie2, vWF, CD31, and VE-cadherin) when they were cultured in endothelial induction medium. Partially agreeing with their results, our data showed that EC-differentiated MSCs were not sufficient to transform into mature endothelial cells because of their very low expression levels of CD31. However, endothelial differentiation did prime MSCs by upregulating some endothelial-related genes and developing endothelial-special functions, such as eNOS generation and acLDL-uptaking capacity, which was in accordance with the study by Janeczek Portalska et al. [37]. Although all of the cells were cultured with endothelial differentiation medium under the same condition, an inconsistent change of endothelial-related genes was seen in our results, which might be due to the wide interdonor variability. A similar result was also seen in the study of Kim et al. [18]. Because the shear stress [38] as well as the small molecule or microgravity [39] could enhance the efficiency of MSC endothelial differentiation, a modified protocol might help MSCs transform into endothelial cells in vitro.

In-vivo MSC transplantation studies demonstrated that very few engrafted MSCs remained several weeks post transplantation. Wu et al. [19] reported that 27 % of transplanted MSCs engrafted into the wounded skin but only 2.5 % cells remained 28 days post administration. Moon et al. [40] demonstrated that less than 1 % engrafted AMSCs were incorporated into the host vascular structures 4 weeks after cell transplantation. In view of the clinical application of MSCs, endothelial differentiation may not be the principal mechanism of MSC-mediated regeneration. Meanwhile, accumulating evidence suggested that the bioactive secretion of MSCs was the dominant reason for their therapeutic benefits [26]. Kwon et al. [33] demonstrated that the CM of human MSCs significantly improved the in-vitro angiogenic activity of endothelial cells and promoted the blood restoration in mice with hind-limb ischemia. Consistently, our result also confirmed the strong proangiogenic action of MSCs via in-vitro paracrine assays.

In addition, many studies have indicated heterogeneous angiogenic gene expression in different MSC types. For example, Hsiao et al. [25] demonstrated a similar mRNA expression level of *VEGF-A*, *bFGF*, *HGF*, and *NGF* in BMSCs and AMSCs. Amable et al. [34] reported that differential secretion was found in BMSCs, AMSCs, and Wharton’s Jelly-derived MSCs, which was consistent with our result, especially on VEGF and HGF secretion. Similarly, Rehman et al. [41], Kandel and Pittenger [42], and Bronckaers et al. [26] also demonstrated a varying paracrine action in different tissue-derived MSCs. Here, our study determined a distinct angiogenic cytokine expression by different MSC types, particularly the secretion of VEGF, HGF, and PGE2. Previous studies have shown that VEGF [43], HGF [44], and PGE2 [32] were important angiogenic cytokines that could significantly promote the growth and tube formation of endothelial cells. Because the different paracrine actions may lead to distinct therapeutic outcomes, some tissue-derived MSC populations with potent paracrine activity should be preconsidering cell banking and clinical application. Our study showed heterogeneous paracrine behaviors of different MSC types, which may indicate potential for tissue source selection of MSCs for clinical therapeutic angiogenesis. Nevertheless, the in-vivo pro-angiogenic properties of different MSC types still need further exploration.

Our FACS results showed distinct vascular cell adhesion molecular-1 (VCAM-1) expression on different MSC types. VCAM-1, also known as CD106, is extensively expressed on endothelial cells [45] and some stromal cells in a particular niche, such as the vascular niche [46] and the hematopoietic niche [47, 48]. Wang et al. [49] described that MSCs pretreated with cytokines (IL-1β and TNF-α) upregulated VCAM-1 expression and revealed an improved treatment effect on cardiovascular ischemia. Recently, our group reported that VCAM-1+ placental chorionic villi-derived MSCs secreted abundant angiogenic cytokines and displayed therapeutic angiogenesis on hind-limb ischemia nude mice [50]. These studies indicated a possible correlation between a higher VCAM-1 expression ratio on MSCs and a better proangiogenic activity. Consistently, our present study suggested that a better paracrine action existed in MSC types with a higher VCAM-1 expression level, such as PMSCs and BMSCs.

In addition, BMSCs, AMSCs, UMSCs, and PMSCs are respectively isolated from different in-vivo environments: bone marrow and placental chorionic villi are abundant with capillaries, adipose tissue is filled with adipocytes, and umbilical cord contains not only blood vessels but also Wharton’s Jelly (the tissue source of UMSCs). These distinct in-vivo environments may affect the bioactivities of MSCs. Konig et al. [51] demonstrated that placental MSCs isolated from blood vessels were better than those from avascular tissues on supporting endothelial cell functions. Jeon et al. [52] reported that placenta-derived MSCs retained a higher therapeutic efficacy than BMSCs and AMSCs in the hind-limb ischemic disease model. Moreover, our RT-PCR results showed a higher angiogenic cytokine expression in the same donor-derived PMSCs rather than UMSCs, which further indicated that the in-situ environment but not the development status might have some effect on the proangiogenic features of MSCs. On the other hand, the in-vitro angiogenic features of MSCs may reflect the characteristics of their different in-situ environment to some extent.

## Conclusions

In this study, we described the heterogeneous proangiogenic features of MSCs derived from different tissue sources, and further demonstrated that the in-vivo environment but not the development status might contribute to their functional heterogeneity. Moreover, our study suggested BMSCs and PMSCs might be preferred in clinical application for vascular diseases due to their potent paracrine actions.

## Additional file

Additional file 1: Table S1. Primers for real-time PCR were listed as follows. (DOCX 1460 kb)

## Abbreviations

acLDL: Acetylated-low density lipoprotein
AMSC: Adipose tissue-derived MSC
ANG: Angiogenin
ANOVA: Analysis of variance
bFGF: Basic fibroblast growth factor
BMSC: Bone marrow-derived MSC
CM: Conditioned medium
Cox-2: Cyclooxygenase-2
EGF: Epidermal growth factor
eNOS: Endothelial nitric oxide synthase
FBS: Fetal bovine serum
HGF: Hepatocyte growth factor
HLA: Human leukocyte antigen
HUVEC: Human umbilical vein endothelial cell
IBMX: 3-Isobutyl-1-methylxanthine
NGF: Nerve growth factor
P/S: Penicillin/streptomycin
PBMC: Peripheral blood mononuclear cell
PFA: Paraformaldehyde
PGE2: Proinflammatory prostaglandin E2
PMSCs: Placental chorionic villi-derived MSCs
TGF-β: Transforming growth factor beta
UMSC: Umbilical cord-derived MSC
VE-Cadherin: Vascular endothelial cadherin
VEGF: Vascular endothelial growth factor
vWF: von Willebrand Factor
