# Supplementary material for: Heterogeneity of proangiogenic features in mesenchymal stem cells derived from bone marrow, adipose tissue, umbilical cord, and placenta
Source: Stem Cell Res Ther. 2016 Nov 10;7:163. doi: 10.1186/s13287-016-0418-9 (PMC5103372; doi:10.1186/s13287-016-0418-9)
Supplement: Additional file 1: Table S1. — Primers for real-time PCR were listed as follows. (DOCX 1460 kb) [file 13287_2016_418_MOESM1_ESM.docx]

**Heterogeneity of pro-angiogenic features in Mesenchymal stem cells derived from Bone Marrow, Adipose tissue, Umbilical Cord and Placenta**

Wen-Jing Du^1†^, Ying Chi^1†^, Zhou-Xin Yang^1^, Zong-Jin Li^2^, Jun-Jie Cui^1^, Bao-Quan Song^1^, Xue Li^1^, Shao-Guang Yang^1^, Zhi-Bo Han^1*^ and Zhong-Chao Han^1, 2*^

^1^ The State Key Laboratory of Experimental Hematology, Institute of Hematology and Hospital of Blood Disease, Chinese Academy of Medical Science & Peking Union Medical College, Tianjin, China; ^2^Beijing Institute of Health and Stem Cells, Beijing, China;

**Supplementary Methods and Materials**

**Flow Cytometry**

Passage 4~6 of BMSCs, AMSCs, UMSCs and PMSCs were collected and their surface antigens were evaluated by flow cytometry. Cells were incubated with antibodies for 30 minutes on ice, including PE-conjugated CD166, CD144, CD133, CD106, CD105, CD73, CD29, and FITC-conjugated CD90, CD45, CD31, HLA-ABC and HLA-DR. PE or FITC conjugated IgG isotype was used as the control. Antibodies were purchased from BD Pharmingen (San Diego, CA, USA). Cells were fixed with 1% paraformaldehyde (PFA, Sigma), and the phenotype was tested by LSRII flow cytometer (BD Bioscience, San Jose, CA, USA). FlowJo 7.6 software (San Carlos, CA, USA) was used to analyze the data.

**Tri-lineage differentiation *in vitro***

To identify the tri-lineage differentiation capacity, BMSCs, AMSCs, UMSCs and PMSCs of passage P3~P5 were harvested and induced into adipocytes, osteocytes, chondrocytes *in vitro*, respectively.

**Adipogenic differentiation:** Cells were cultured in adipogenic differentiation medium for three weeks *in vitro*. The complete adipogenic differentiation medium contained DF12 medium, 10%FBS, 100U P/S, 2mM glutamine, 1µM dexamethasone (Sigma), 0.5 mM 3-isobutyl-1-methylxanthine (IBMX; Sigma), 10µM insulin (Sigma), and 100µM indomethacin (Sigma). The culture medium was changed twice a week. Oil Red O staining was used to test the efficiency of adipogenic differentiation.

**Osteogenic differentiation:** Cells were cultured in osteogenic differentiation medium for 3 weeks *in vitro*, and then MSC-derived osteocytes were further assessed by staining with von Kossa and Alizarin Red. The osteogenic differentiation medium contained DF12, 10% FBS, 100U P/S, 2mM glutamine, 0.1µM dexamethasone, 10mM β-glycerophosphate (Sigma) and 0.2mM ascorbic acid (ASA, Sigma).

**Chondrogenic differentiation:** At least in 10^6^ MSCs were harvested and centrifuged at 160g for 8 minutes. Then cell pellets were respectively cultured with 10ml chondrogenic differentiation medium in 15ml centrifuge tubes at 37°C. The chondrogenic differentiation medium contained high glucose DMEM (Gibco), 10ng/ml transforming growth factor (TGF)-β1 (Prepotech), 0.1µM dexamethasone, 50µg/ml ASA, 100µg/ml sodium pyruvate (Sigma), 40µg/ml proline (Sigma), 50mg/ml Insulin-Transferrin-Selenium-A (6.25µg/ml insulin, 6.25µg/ml transferring, 6.25ng/ml selenious acid; Gibco, Auckland, NZ), 1.25mg/ml albumin bovine V (Solarbio), and 5.35mg/ml linoleic acid (Sigma). Cell pellets were conducted chondrogenic differentiation *in vitro* for three weeks, and the medium was changed twice a week. When the chondrogenic differentiation completed, cell pellets were collected, fixed with 4%PFA, embedded in Tissue-Tek O.C.T compound (Sakura Finetek USA, Inc., Torrance, CA, USA) at -20°C and made frozen slices. Later, frozen slices were fixed with ice-acetone (sigma) for 15minutes, and kept at -20°C. Alcian Blue staining was performed to evaluate the chondrogenic differentiation of MSCs, and photos were taken at ×100 magnification.

**Supplementary Results**

**Characteristics of BMSCs, AMSCs, UMSCs and PMSCs**

BMSCs, AMSCs, UMSCs and PMSCs were cultured and passaged in vitro. MSCs regardless of their tissues origins all appeared fibroblast-like shapes. FACS analysis was used to study the phenotype of BMSCs, AMSCs, UMSCs and PMSCs, and results were shown in **Supplemental** **Fig.1 and Supplement Table 2 (S2)**. The result indicated that MSCs derived from bone marrow, adipose tissue, umbilical cord, and placental chorionic villi shared similar surface markers, i.e. they were positively expressed CD105, CD73, CD90, CD29, CD166 and HLA-ABC, absent of hematopoietic markers CD45, immunogenic indicator HLA-DR and early or late stage of endothelial cell markers CD133, CD31 and CD144. Additionally, the endothelial or mesenchyme marker CD106 was expressed varied among BMSCs, AMSCs, UMSCs and PMSCs [1].

To investigate the tri-lineage differentiation, BMSCs, AMSCs, UMSCs and PMSCs were differentiated into adipocytes, osteocytes, and chondrocytes in vitro, respectively. After three weeks adipogenic differentiation, Oil Red O staining was performed to stain the lipid droplets. We found adult tissue-derived BMSCs and AMSCs were more inclined to form lipid droplets with comparison to perinatal tissue-derived UMSCs and PMSCs **(Supplemental** **Fig.2A).** Additionally, Alizarin Red **(Supplemental** **Fig.2B)** and von Kossa **(Supplemental** **Fig.2C)** staining were used to assess the osteogenic differentiation of BMSCs, AMSCs, UMSCs, and PMSCs. The mineralized extracellular matrix generated from osteocytes could be stained with alizarin red and von kossa. Our data showed both adult and perinatal tissue-derived MSCs were capable of osteogenic differentiation. Besides, Alcian blue staining was performed to stain the sulfate glycosaminoglycan matrix deposition of chondrocytes. After three weeks chondrogenic differentiation, BMSCs, AMSC, UMSCs, and PMSCs successfully transformed into chondrocytes which positively stained with Alcian blue (**Supplemental** **Fig.2D**). Referring to the minimum criteria of MSCs [2], perinatal tissue-derived UMSCs and PMSCs fitted well with the definition of MSCs as BMSCs and AMSCs.

**Supplemental** **Tables**

**Table S1: Primers for real-time PCR were listed as follows.**

| Genes | Forward 5’→3’ | Reverse 5’ →3’ |
| --- | --- | --- |
| *CD31* | AACAGTGTTGACATGAAGAGCC | TGTAAAACAGCACGTCATCCTT |
| *CD34* | ACCAGAGCTATTCCCAAAAGACC | TGCGGCGATTCATCAGGAAAT |
| *Flt-1* | ATGGAAAACGCATAATCTGGGAC | AAATGCCCATTGACTGTTGCT |
| *vWF* | CCTTGACCTCGGACCCTTATG | GATGCCCGTTCACACCACT |
| *VE-Cadherin* | AAGCGTGAGTCGCAAGAATG | TCTCCAGGTTTTCGCCAGTG |
| *Tie-2* | CAGGATACGAACCATGAAGATGC | GGGGCACTGAATGGATGAAG |
| *VEGF-A* | AGGGCAGAATCATCACGAAGT | AGGGTCTCGATTGGATGGCA |
| *VEGF-C* | GGCTGGCAACATAACAGAGAA | CCCCACATCTATACACACCTCC |
| *bFGF* | AGTGTGTGCTAACCGTTACCT | ACTGCCCAGTTCGTTTCAGTG |
| *HGF* | GCTATCGGGGTAAAGACCTACA | CGTAGCGTACCTCTGGATTGC |
| *ANG* | CAAGGCCATCTGTGAAAACAAG | CAGGGGGAACCTCCATGTAG |
| *NGF* | GGCAGACCCGCAACATTACT | CACCACCGACCTCGAAGTC |
| *IL-1α* | CGCCAATGACTCAGAGGAAGA | GCAGCAGCCGTGAGGTACT |
| *IL-1β* | AGCTACGAATCTCCGACCAC | CGTTATCCCATGTGTCGAAGAA |
| *IL-6* | CCACACAGACAGCCACTCAC | CCAGATTGGAAGCATCCATC |
| *IL-8* | TTGGCAGCCTTCCTGATTT | TCAAAAACTTCTCCACAACCC |
| *TGF-β* | ACTGCAAGTGGACATCAACG | TGCGGAAGTCAATGTACAGC |
| *Cox2* | ACTCTGGCTAGACAGCGTAA | ACCGTAGATGCTCAGGGAC |

**Table S2: FACS analysis of phenotype of BMSCs, AMSCs, UMSCs and PMSCs.**

Data were presented as Mean ± S.E.M.

| Antibodies (%) | BMSCs  （n=3） | AMSCs  （n=3） | UMSCs  （n=3） | PMSCs  （n=3） |
| --- | --- | --- | --- | --- |
| CD105 | 84.5±3.8 | 96.5±1.6 | 98.4±0.4 | 93.9±1.2 |
| CD73 | 93.6±2.6 | 98.7±0.3 | 99.5±0.2 | 98.4±0.2 |
| CD90 | 85.5±5.3 | 96.9±1.7 | 96.0±2.3 | 82.3±7.9 |
| CD29 | 92.4±5.6 | 98.2±0.5 | 99.5±0.2 | 97.8±0.8 |
| CD166 | 50.0±14.1 | 82.5±11.2^*^ | 90.4±4.3^#^ | 88.3±6.4^+^ |
| CD106 [1] | 13.0±10.5 | 0.2±0.2^&^ | 4.0±2.1^^^ | 68.2±7.9^+^ |
| CD45 | 0.4±0.3 | -0.3±0.2 | -0.2±0.02 | 0.5±0.6 |
| HLA-ABC | 75.2±3.9 | 93.1±3.3^*^ | 98.6±0.5^#^ | 94.3±2.1^+^ |
| HLA-DR | 1.8±0.8 | -0.3±0.2 | 0.3±0.2 | 0.1±0.1 |
| CD144 | 0.1±0.3 | -0.2±0.3 | 0.5±0.7 | 1.7±1.9 |
| CD31 | 0.5±0.4 | 0.0±0.1 | 0.5±0.4 | 0.2±0.2 |
| CD133 | 0.3±0.4 | -0.3±0.1 | -0.1±0.2 | -0.1±0.1 |

^*^ indicated BMSCs vs AMSCs showed different significantly (*p<0.05*). Similarly,

^#^ indicated BMSCs vs UMSCs (*p<0.05*); ^+^ indicated BMSCs vs PMSCs (*p<0.05*);

^&^ indicated AMSCs vs PMSCs (*p<0.05*); ^^^ indicated UMSCs vs PMSCs (*p<0.05*).

**Supplemental Figures:**

**Supplemental Fig.1: BMSC, AMSC, UMSC and PMSC share similar surface markers.** The data was presented as the mean percentage of positive cells (n=3). Typical MSC surface markers include CD105, CD90, CD73, CD29 and CD166. Immunogenic markers: HLA-ABC and HLA-DR; hematopoietic and endothelial cells related markers: CD45, CD144, CD133, and CD31; Mesenchymal/Endothelial marker: CD106. Three donors derived MSCs were used.


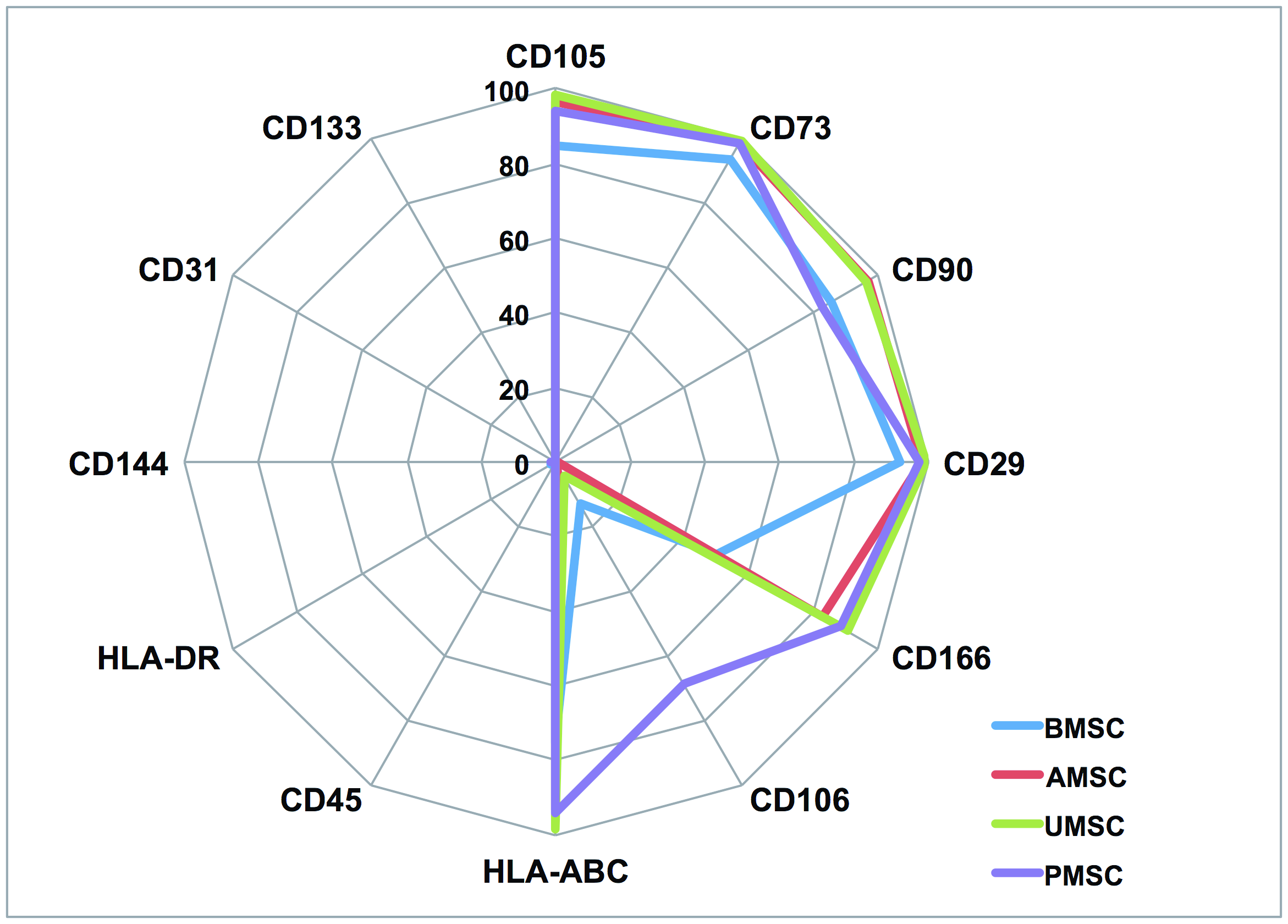


**Supplemental Fig.2: Tri-lineage differentiation of BMSC, AMSC, UMSC and PMSC.** MSCs cultured in specific differentiation medium for three weeks displayed their adipogenic, osteogenic and chondrogenic commitment. (A) Adipogenic differentiation was verified by Oil Red O staining (scale bar=100μm). The osteogenic transformation was confirmed by staining with Alizarin Red (B) and von Kossa (C) (scale bar=200μm or 500μm). (D) Alcian Blue staining was performed to identify the sulfated glycosaminoglycan matrix deposition in chondrocytes trans-differentiated from BMSC, AMSC, UMSC and PMSC (scale bar=200μm).


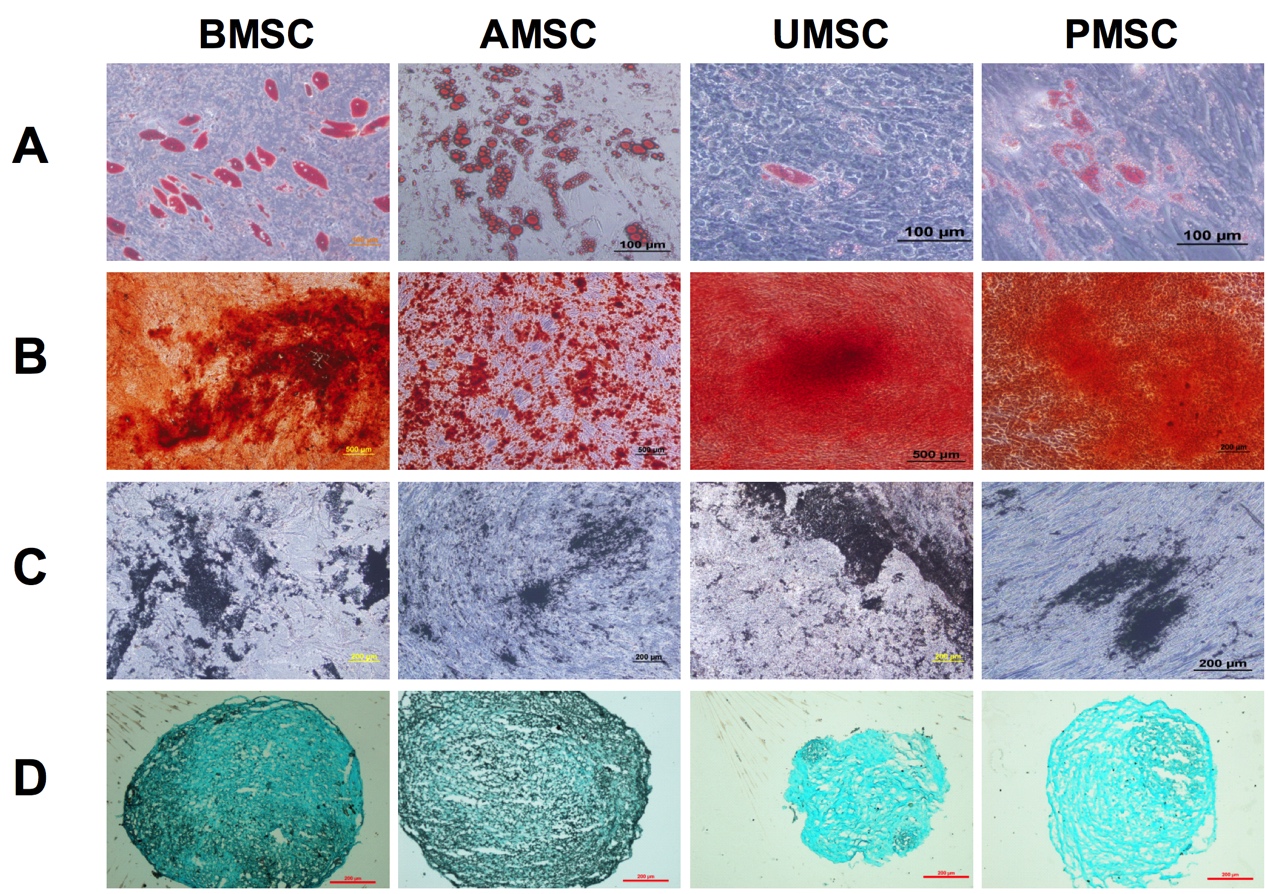


**Reference:**

1. Yang, Z.X., et al., *CD106 identifies a subpopulation of mesenchymal stem cells with unique immunomodulatory properties.* PLoS One, 2013. **8**(3): p. e59354.

2. Dominici, M., et al., *Minimal criteria for defining multipotent mesenchymal stromal cells. The International Society for Cellular Therapy position statement.* Cytotherapy, 2006. **8**(4): p. 315-7.
